# The westward spread dynamics of cholera from the eastern endemic Democratic Republic of the Congo

**DOI:** 10.1080/22221751.2024.2437245

**Published:** 2024-12-04

**Authors:** Harry César Ntumba Kayembe, Nadège Makuntima Taty, Hippolyte Nani-Tuma Situakibanza

**Affiliations:** a One Health Institute for Africa, Faculty of Medicine, University of Kinshasa, Kinshasa, Democratic Republic of the Congo; b National Program for the Elimination of Cholera and the Control of Other Diarrheal Diseases, Ministry of Health, Hygiene and Prevention, Kinshasa, Democratic Republic of the Congo; c Department of Internal Medicine, Faculty of Medicine, University of Kinshasa, Kinshasa, Democratic Republic of the Congo

## Main text

Cholera remains a significant global public health threat. Notwithstanding the fact that this acute and severe diarrheal illness is both preventable and treatable, it is estimated that millions of cases and thousands of deaths are attributed to it each year [1,2]. Sub-Saharan Africa is one of the regions with the highest cholera burden [3,4]. The Democratic Republic of Congo (DRC), situated at the heart of the African continent, has been grappling with significant cholera epidemics for several years, making it one of the countries facing the longest-standing cholera crisis on the continent and globally [1,2,4]. Since the beginning of 2024, the ongoing cholera epidemic in the DRC has resulted in over 25,000 suspected cases and more than 350 deaths [5].

At the subnational level, large-scale and recurrent epidemics have been repeatedly documented around the Great Lakes Region (GLR) in eastern DRC [6,7]. Genomic analyses have confirmed that the eastern provinces bordering the lake basins of the African Rift Valley are the source of the epidemics that have been reported in the western part of the DRC [8]. Consequently, the lakeside areas of the GLR, which are considered cholera hotspots, serve as a point of origin for epidemics and their subsequent dissemination to other vulnerable regions [9]. These areas play a central role in the dynamics of cholera propagation in the country.

In a recent publication, Irenge et al. presented phylogenetic characteristics of *V. cholerae* isolates associated with cholera outbreaks in three eastern DRC provinces (North Kivu, South Kivu, and Tanganyika) and one central DRC province (Kasai Oriental) from 2018 to 2022 [10]. This study follows a previous genomic analysis of isolates from the eastern DRC between 2014 and 2017 [11]. They demonstrated that the *V. cholerae* O1 isolates from Kasai Oriental province are phylogenetically linked to isolates from GLR provinces. This finding lends further support to the hypothesis that cholera spread westward in the DRC. Moreover, the authors have indicated that this dynamic of geographic cholera expansion is inconsistent with other hypotheses on potential transmission routes in central DRC, published in 2021 [12].

**Figure 1.**
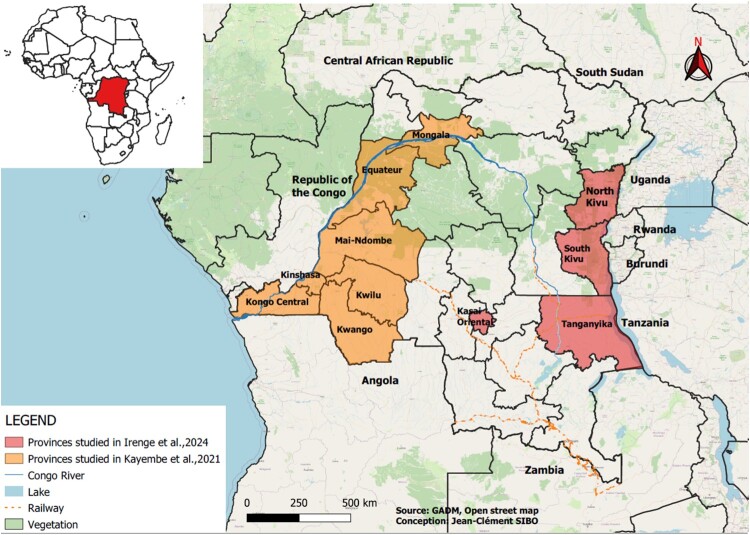
Provinces included in Irenge et al. [10] and Kayembe et al. [12].

**Figure 2.**
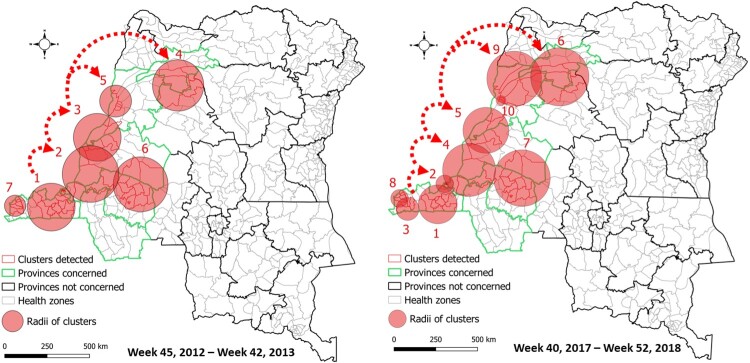
Spatiotemporal clusters of cholera cases and main likely routes of epidemic spread from downstream to upstream of the Congo River in western DRC.

Unfortunately, this assertion does not accurately represent the contextual framework of the analytical outcomes presented in the aforementioned publication [12]. Firstly, the provinces studied in our research do not overlap with the provinces studied in Irenge et al. [10] (Figure 1). Consequently, the conclusions of the two studies are not mutually exclusive, as they do not study the same provinces over the same time periods. Secondly, the main objective of our study was to investigate the major epidemics of cholera that affected the western region of the DRC. Through a comprehensive literature review of historical geography and genomic evidence [12], it was determined that the initial cholera cases in the DRC were introduced into the country's southwestern region in 1973. Thirdly, with regard to epidemics that have spread from the endemic eastern region, the study is based on the hypothesis that there are propagation dynamics other than the unilateral east–west model along the Congo River, from upstream to downstream. Accordingly, an examination of the spatio-temporal progression of the disease, coupled with the successive identification of significant clusters of cases from the country's westernmost region (southwest) to areas in the northern provinces, serves to underscore complementary patterns of epidemic expansion from downstream to upstream of the Congo River (Figure 2). These patterns are superimposed on the western extension dynamics of cholera. Additionally, the spread dynamics of cholera along main rivers have also been documented across the African continent [13].

**Figure 3.**
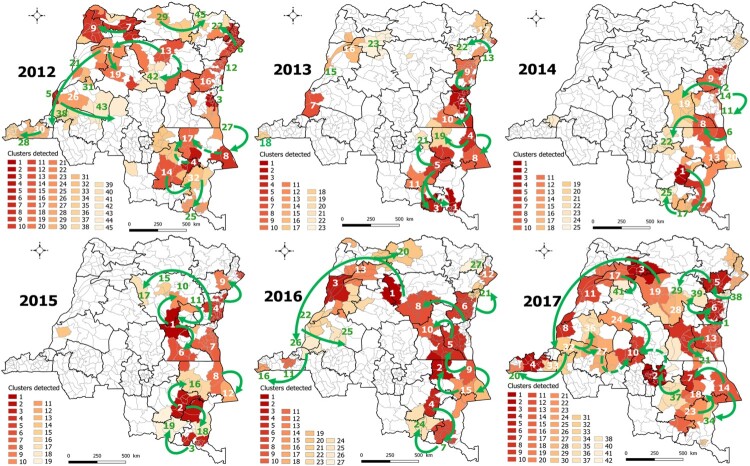
Spatiotemporal clusters of cholera cases and main likely routes of epidemic spread from the eastern endemic DRC.

Nevertheless, it is important to note that the results of a study published in 2022 are entirely consistent with the evidence indicating a westward expansion dynamic of cholera and possible transmission routes from the GLR provinces to other regions, particularly central DRC (Figure 3) [14]. As suggested by Irenge et al. [10], the lake areas of the endemic eastern provinces would serve as the primary source of epidemics affecting the eastern portion of central DRC, including Kasai Oriental, via the railways [14,15].

In summary, the propagation of cholera epidemics follows preferential trajectories, which illustrate their predictive nature [12,14]. The consideration of these preferential trajectories in the early warning, alert, and response system of integrated cholera surveillance can facilitate the anticipation of the risk of outbreak expansion in the DRC, as well as in other countries with a high incidence of the disease.
